# Arthroscopic Electromechanical Assessment of Human Articular Cartilage Injury Correlates with ICRS Scores

**DOI:** 10.1177/19476035231216439

**Published:** 2023-12-06

**Authors:** Augustinas Rimkunas, Rimtautas Gudas, Tomas Mickevicius, Justinas Maciulaitis, Mantas Malinauskas, Alfredas Smailys, Mantas Staskunas, Arvydas Usas

**Affiliations:** 1 Lithuanian University of Health Sciences, Medical Academy, Faculty of Medicine, Department of Orthopaedics and Traumatology, Kaunas, Lithuania; 2 Lithuanian University of Health Sciences, Medical Academy, Faculty of Medicine, Institute of Physiology and Pharmacology, Kaunas, Lithuania

**Keywords:** arthroscopic cartilage evaluation, electromechanical properties, ICRS grade, cartilage injury

## Abstract

**Purpose:**

This study aimed to conduct arthroscopic evaluation of cartilage electromechanical properties and establish their correlation with International Cartilage Repair Society (ICRS) grading scores.

**Methods:**

In 18 patients, quantitative parameter (QP) measurements were taken on the weight-bearing surface of the medial femoral condyle. Adjacently, the same site was graded using ICRS scores (0-4). Electromechanical QPs for ICRS grades 0 to 3 were obtained during arthroscopy, while complete grade 4 injuries were assessed using femur cartilage-bone blocks from knee arthroplasty. The QP values for ICRS grades 0 to 2 were compared with grades 3 and 4 using Welch *t* test. The corresponding QP values were assigned to ICRS grades 0 to 4 and compared using Welch ANOVA (analysis of variance). Pearson’s coefficient evaluated QP-ICRS grade relationship.

**Results:**

Healthy grade 0 cartilage displayed a mean QP value of 10.5 (±2.8 SD, *n* = 4). The ICRS grade 1 and grade 2 injuries were associated with QP values of 12 (±0.7, *n* = 2) and 13.25 (±1.77, *n* = 2), respectively. The grade 3 defects had QP values of 20.43 (±4.84, *n* = 4), whereas complete grade 4 defects showed electromechanical values of 30.17 (±2.19, *n* = 6). Significant differences in QP values were observed between ICRS grades 0 to 2 (mean QP 11.56 ± 2.3, *n* = 8) and grades 3 and 4 (26.27 ± 6, *n* = 10; *P* < 0.0001). Pearson’s correlation coefficient of 0.9 indicated a strong association between higher ICRS cartilage injury grades and elevated QP values (*P* < 0.0001).

**Conclusion:**

Arthroscopic electromechanical QP assessment robustly correlates with ICRS scores. The QP values for ICRS grades 0 to 2 are significantly lower, compared with grades 3 and 4.

## Introduction

Along with the aging population and increasing obesity rates, the number of cases of degenerative articular cartilage pathology is growing exponentially.^1^ In addition, greater number of traumatic knee injuries leads to increased frequency of cartilage disease.^1,2^ The problem is evident in the growing number of arthroscopic knee surgeries year by year.^3^ Arthroscopies are performed for focal cartilage reconstruction, to treat meniscus, and ligament ruptures to prevent osteoarthritis progression, while an articular cartilage damage is an integral part of most of these illnesses. Therefore, precise preoperative and intraoperative articular cartilage evaluations remain a crucial challenge for orthopedic surgeons.

Currently, one of the most accurate articular cartilage assessment methods is MRI. Although cartilage-specific MRI sequences are sensitive up to 83% compared with arthroscopic evaluation, clinical MRI remains even more inaccurate in detecting cartilage lesions.^4,5^ Defects, cracks, and thinning can be observed on MRI-visualized cartilage, although images do not represent collagen and proteoglycan loss in the tissue, which alters cartilage function. Arthroscopic assessment is considered as a “gold standard” to evaluate cartilage lesions.^4,6^ While MRI remains useful for preoperative planning, routinely during any arthroscopic procedure the cartilage is reevaluated by investigating the surface with the blunt arthroscopic probe. The surgeon examines the texture and integrity of the cartilage, while by applying slight pressure the elasticity and stability are assessed. Based on findings and visual appearance, the cartilage is graded using International Cartilage Repair Society (ICRS) score. Despite clinically used indirect radiological and subjective intraoperative cartilage evaluation methods, there is a great demand for an objective cartilage evaluation technique.

One of the alternative evaluation methods is nondestructive electromechanical articular cartilage assessment using Arthro-BST device (Biomomentum, Laval, Quebec, Canada). The rationale of using electromechanical cartilage evaluation method is based on streaming potentials, which are pressure-induced electric potentials resulting from the interaction between solid and liquid mediums of the cartilage. The extracellular matrix (ECM) of the cartilage is considered as a solid medium, consisting of type 2 collagen and the sulfated proteoglycans, mostly aggrecan. Large proteoglycan molecules are entrapped in the fibrillar web of type II collagen in association with type XI and IX collagens.^7^ Aggrecan, with numerous negatively charged chondroitin sulfate glycosaminoglycan (GAG) chains, has high affinity for interstitial fluid rich in positive ions. Negatively charged GAG chains draw water and sodium into the tissue.^7^ Water and positively charged sodium, potassium, and calcium ions in the tissue are perceived as a liquid cartilage medium.^8^ In equilibrium condition without load, no electric field exists due to symmetrical arrangement of mobile cations around the negatively charged ECM.^9^ Under cartilage surface pressure, mobile positively charged ions move out of the tissue against fixed negatively charged matrix molecules, creating measurable streaming potentials.^8^ Using a special probe containing 37 microelectrodes on the Arthro-BST indenter during instantaneous cartilage pressure, the device registers streaming potentials. A nonplanar microelectrode array is used to monitor indenter-cartilage contact and measure streaming potentials.^10^ The manufacturer’s software converts streaming potentials to numeric values—quantitative parameters (QP) that reflect biochemical and mechanical properties of the cartilage.^11^ Depending on cartilage biochemical quality, different QPs ranging from 0 to 36 are generated. Due to traumatic or degenerative changes, the cartilage ECM is damaged and proteoglycan and collagen molecules are lost, leading to altered streaming potentials. The greater the ECM loss, the higher the QP values are generated by the device.^12,13^ The reliability of the technique has been widely tested on animal specimens although only a few studies report results of intraoperative evaluation of human articular cartilage.^14^

The aim of this study was to evaluate the correlation of electromechanical QP measurements obtained during knee surgeries by comparing them with widely clinically used ICRS cartilage injury evaluation scores. In addition, it was sought to determine the difference between lower ICRS grades 0 to 2 scores, which do not require surgical reconstruction, and greater ICRS 3 and 4 grades, when cartilage reconstruction is indicated, and to compare the differences between individual ICRS grades.^15^ Moreover, we aimed to establish the guidelines of approximate QP values that could be attributed to normal and injured cartilage ICRS scores that can serve as a reference point for further research comparison and clinical evaluation.

## Materials and Methods

### Ethics and Criteria for Inclusion in the Study

The study was approved by the bioethics committee at the author’s institution (no. BEC-MF-243). All patients signed informed consent forms. Electromechanical cartilage evaluation was performed on 18 patients who underwent knee surgery due to various pathologies. Patients with concomitant diseases, such as diabetes or inflammatory joint disease, which could disrupt cartilage homeostasis and distort the results, were excluded.

## Macroscopic Assessment

Macroscopically articular cartilage lesions were graded using ICRS classification that is still widely used up to date.^16-18^ The classification is based on visual cartilage appearance and depth of the defect. According to the ICRS classification, grade 0 represents normal healthy cartilage, whereas grades 1 and 2 define minor cartilage changes. Superficial lesions, including soft indentation, fissures, and cracks are classified as grade 1, whereas lesions extending down less than 50% of the depth are classified as grade 2. Severe cartilage changes are classified as grades 3 and 4. Defects extending down more than 50% of cartilage depth and down to the calcified layer up to subchondral bone are classified as grade 3, whereas full thickness cartilage defects extending through subchondral bone are classified as grade 4.

## Electromechanical Evaluation

**Figure 1.**
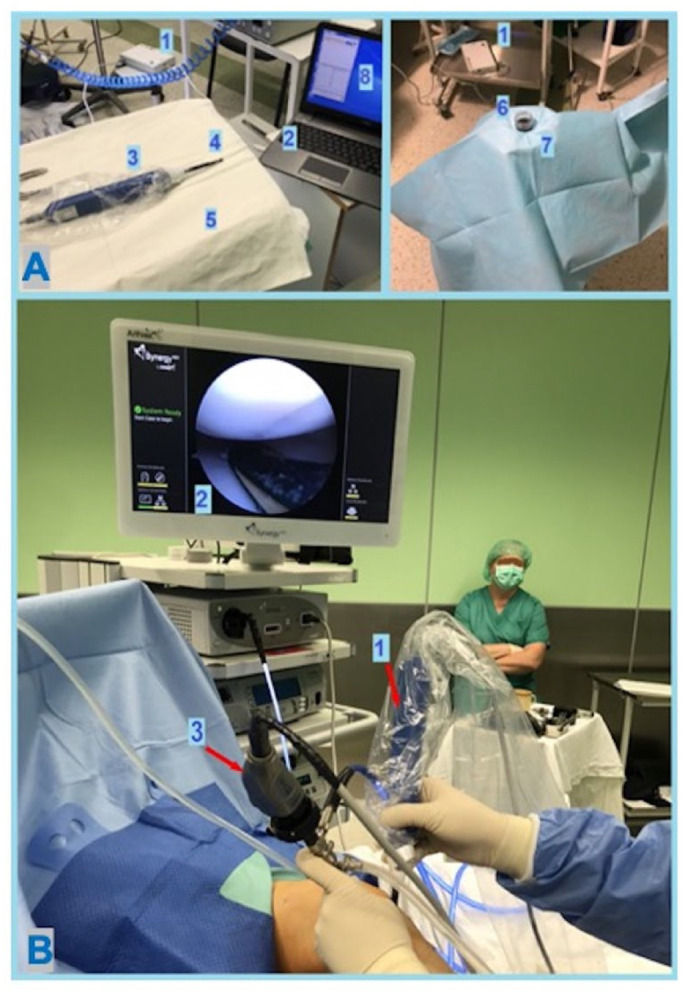
Electromechanical evaluation during knee arthroscopic surgery. (**A**) Preparation for electromechanical measurements. (1) Arthro-BST electrical isolation box. (2) Laptop computer. (3) Arthro-BST handle. (4) Sterile tip with electrodes. (5) Sterile table. (6) Sterile container with saline. (7) Sterile-covered ultrasound bath. (8) Arthro-BST software. (**B**) Electromechanical assessment during arthroscopy. (1) Sterile draped Arthro-BST handle with attached tip. (2) Sterile tip with electrodes at the measurement site of medial femoral condyle weight-bearing surface. (3) Regular arthroscopic camera.

The patient was positioned supine with spinal anesthesia. Standard aseptic preparation and draping for arthroscopic surgery was followed by additional draping of the Arthro-BST handle, similar to an arthroscopic shaver, in a sterile fashion. The sterile Arthro-BST tip was opened and inserted into the handle. The handle was connected to the computer software and Arthro-BST isoelectric box. A sterile ultrasound container was filled with 30 mL of sterile normal saline fluid (0.9% sodium chloride) for the calibration of the device (**Fig. 1A**). Standard medial and lateral parapatellar portals were made for arthroscopic surgery. The joint was inflated using 0.9% sodium chloride solution. Before every measurement, equilibration was achieved by immersing the tip in the ultrasonic container.^19^ After the software displays a straight line with yellow light, the device is calibrated and ready to use. The probe was inserted through medial parapatellar portal. The surgeon gently pressed the microelectrodes of the hemispherical probe to the cartilage. After the technician on the software display observed the drop and rise of the electromechanical line, the “process” button was pressed. The QP value was displayed on the screen and noted. If the probe is mispositioned, a message to repeat the measurement appears with no QP result.^19^

**Figure 2.**
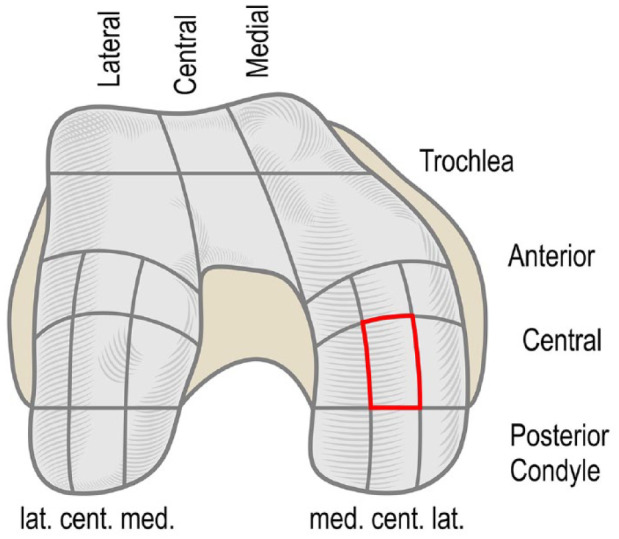
Femoral articular cartilage surface zones. The investigation site, medial femoral condyle central-central weight-bearing zone, is marked red. The weight-bearing region of the medial femoral condyle was visualized by flexing the knee to 45° and applying valgus pressure during arthroscopy.

The QP measurements of the healthy and injured cartilage were performed on standing weight-bearing surface of the medial femoral condyle (**Fig. 1B**). Based on previously described grid system, the QP measurements were taken on medial femoral condyle central-central surface^20^ (**Fig. 2**). The weight-bearing region of the medial femoral condyle was visualized by flexing the knee to 45° and applying valgus pressure during arthroscopy. The central part of the condyle was chosen for measurement. The cartilage injury of interest was on the described femoral site. If the cartilage defect was not limited only to the central-central zone, the measurement was still made at the described region and ICRS grade appointed according to the defect’s magnitude of the central-central zone. Per joints 2 and 3, electromechanical QP measurements were performed, and average values were included in the statistics per patient. The ICRS grading and electromechanical assessments were performed by the same senior orthopedic surgeon.

Complete grade 4 cartilage defects were evaluated using distal femur cartilage-bone blocks removed during knee arthroplasty. A different setting was used as ICRS grade 4 defects are full thickness cartilage defects extending through subchondral bone plate and usually exhibiting clear signs of advanced osteoarthritis. Therefore, it was anticipated that such patients will be seen very rarely in an arthroscopic setting. Moreover, it is not possible to record the QP measurement on the surface of subchondral bone defect with no cartilage layer as there is no cartilage matrix. The former phenomenon was described when relatively healthy range QP values were attributed to ICRS grade 4 cartilage.^13^ Consequently, in this study, ICRS grade 4 measurements were performed on the edge of the defect, where a thin layer of cartilage was still present. To reproduce arthroscopic measurement conditions, the bone blocks were immediately placed in a plastic dish filled with 0.9% sodium chloride solution for evaluation.

## Magnetic Resonance Imaging Evaluation

Magnetic resonance imaging images of one random patient per each ICRS grade were gathered for visual reference to QP values. Examinations were performed at different institutions, using different MRI devices. Patients with ICRS grades 0 and 1 were examined with T2 turbo spin-echo (TSE) imaging sequence, whereas patients having grades 2 and 3 were investigated with PD (Proton Density) TSE. Although all sequences are used for cartilage evaluation, given the former differences, the MRI images are provided solely for visual reference to corresponding electromechanical QP values.

Electromechanically measured zone of medial femoral condyle was evaluated using a traditional Outerbridge scale on MRI.^21^ Outerbridge MRI grade is based on cartilage defect depth on MRI and signal intensity. Outerbridge grade 0 describes healthy cartilage. Bone edema without cartilage damage is scored as grade 0.5. Cartilage signal heterogeneity involved in the score is graded as 1. Outerbridge grade 2 describes degeneration of less than 50% of cartilage thickness, while grade 3 includes more than 50%. Full thickness cartilage defect is described as grade 4. Images were manipulated using OsiriX DICOM Viewer (v. 13.0.1; Pixmeo SARL, Bernex, Switzerland).

## Statistical Analysis

The statistical and analysis unit was 1 patient. Medial femoral condyle zone of the patient was evaluated 2 or 3 times and the average QP value was used. Electromechanical QP values were appointed to 5 groups based on ICRS grades ranging from 0 to 4. GraphPad Prism 9.0 software was used for statistical analysis and figures.

The QP values attributed to ICRS grades of 0 to 2 and ICRS 3 and 4 were compared using Welch *t* test.

A 1-way Welch ANOVA (analysis of variance) with Dunnett’s T3 multiple comparison test was used to compare QP values of individual multiple ICRS groups simultaneously. The statistical significance was expressed as a 2-sided *P* value. The relationship between the QP and ICRS values was assessed by parametric correlation analysis using Pearson’s correlation coefficient “r.” Significance level was set at *P* = 0.05.

## Results

A total of 18 patients were involved in the study. According to ICRS classification, the following cartilage lesions were detected—grade 0 (4 patients, 30 years [30] male (M), surgically treated for ACL (anterior cruciate ligament) tear, 36 years (36) female (F), for both menisci rupture, 28 M, ACL tear, 40 M, medial meniscus rupture, 8 QP measurements), grade 1 (2 patients, 26 M, ACL tear, 37 F, ACL tear, 4 QP measurements), grade 2 (2 patients, 28 M, ACL tear, 56 F, both menisci rupture, 4 QP measurements), grade 3 (4 patients, 25 M, ACL rupture, 44 M, medial meniscus tear, 60 F, treated for focal cartilage defect, 54 F, focal cartilage defect, 8 QP measurements), and grade 4 (6 patients, 66 F, 70 M, 69 F, 79 F, 74 M, 76 F, all treaded for grades 3 and 4 osteoarthritis, 18 QP measurements).

**Figure 3.**
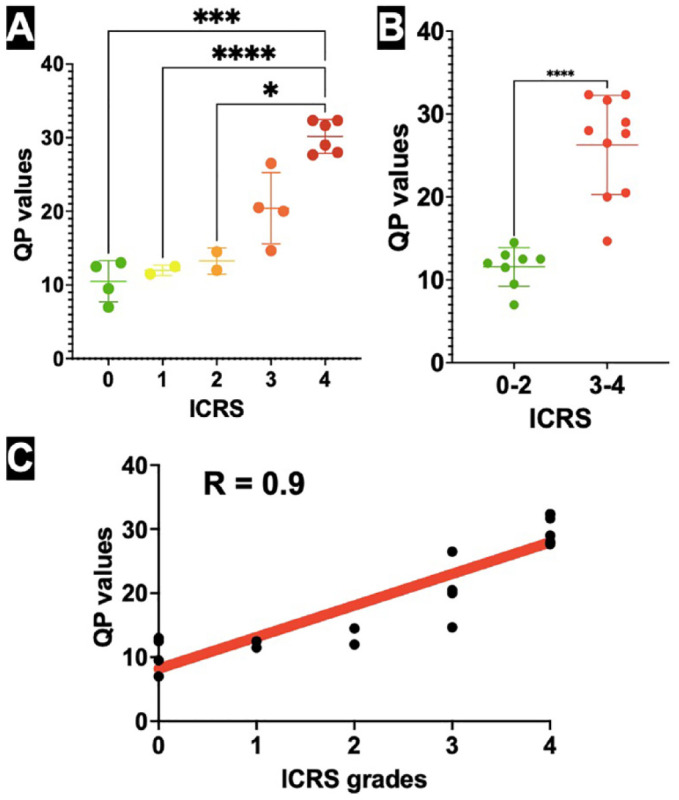
Results. (**A**) A scatter dot plot graph displays individual mean QP values of ICRS grades from 0 to 4, with standard deviation. The simultaneous comparison was made using Welch ANOVA with Dunnett’s T3 correction. The significance level was set at *P* < 0.05. (**B**) A scatter dot plot graph displays mean QP values with standard deviation of ICRS grades 0 to 2 and greater ICRS grades of 3 and 4, requiring surgical reconstruction. The comparison was made using Welch *t* test. The significance level was set at *P* < 0.05. (**C**) Strong positive Pearson’s correlation between higher ICRS grades and greater QP values. R = 0.9, *P* < 0.0001. Medial femoral condyle weight-bearing surface of 18 patients (n = 18); significance level was set at *P* < 0.05. QP = quantitative parameter; ICRS = International Cartilage Repair Society; ANOVA, analysis of variance. An asterisk (*) indicates statistical significance at the 0.05; (***) at the 0.005, while (****) indicates statistical significance at the 0.001.

The mean QP of 10.5 (±2.8 SD) was attributed to healthy grade 0 cartilage, whereas QPs of 12 (±0.7 SD) and 13.25 (±1.77 SD) were appointed to ICRS grade 1 and grade 2, respectively. Defects extending more than 50% of cartilage depth, ICRS grade 3, generated QP values of 20.43 (±4.84 SD); meanwhile, complete grade 4 cartilage defects had electromechanical values of 30.17 QP (±2.19 SD). A box plot graph displays individual values of data sets with standard deviation (**Fig. 3A**).

There was a statistical difference of QP values between lower ICRS grades of 0 to 2 (11.56 ± 2.3 SD), which do not require cartilage reconstruction procedures, and greater ICRS grades 3 and 4 (26.27 ± 6 SD; *P* < 0.0001; **Fig. 3B**). Comparing separate ICRS grades statistically, significant differences of QP values were observed only between ICRS grades 0 to 2 and ICRS grade 4, given uneven sample size among groups (*P* < 0.0005, *P* < 0.0001, and *P* = 0.0307, respectively).

A strong positive correlation was determined between greater ICRS cartilage injury scores and higher QP values; Pearson’s correlation coefficient r = 0.9 (*P* < 0.0001; **Fig. 3C**).

**Figure 4.**
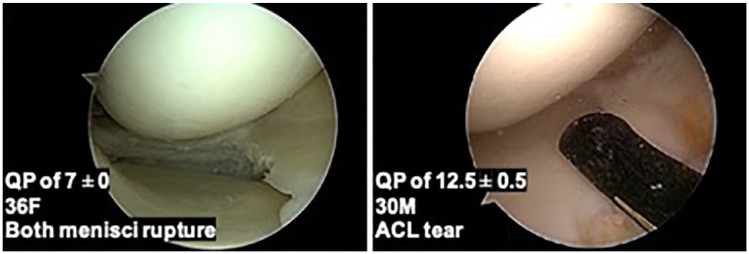
Varying QP values and arthroscopic images of healthy ICRS 0 group patients are represented. A 36-year-old woman treated for both menisci rupture with a QP value (7 ± 0 SD) and a 30-year-old man treated for ACL tear (QP of 12.5 ± 0.5 SD). QP = quantitative parameter; ICRS = International Cartilage Repair Society; ACL = anterior cruciate ligament.

Varying QP values of healthy ICRS 0 group patients are represented; a 36 F treated for both menisci rupture with a QP value (7 ± 0 SD) and a 30 M treated for ACL tear (QP of 12.5 ± 0.5 SD; **Fig. 4**).

**Figure 5.**
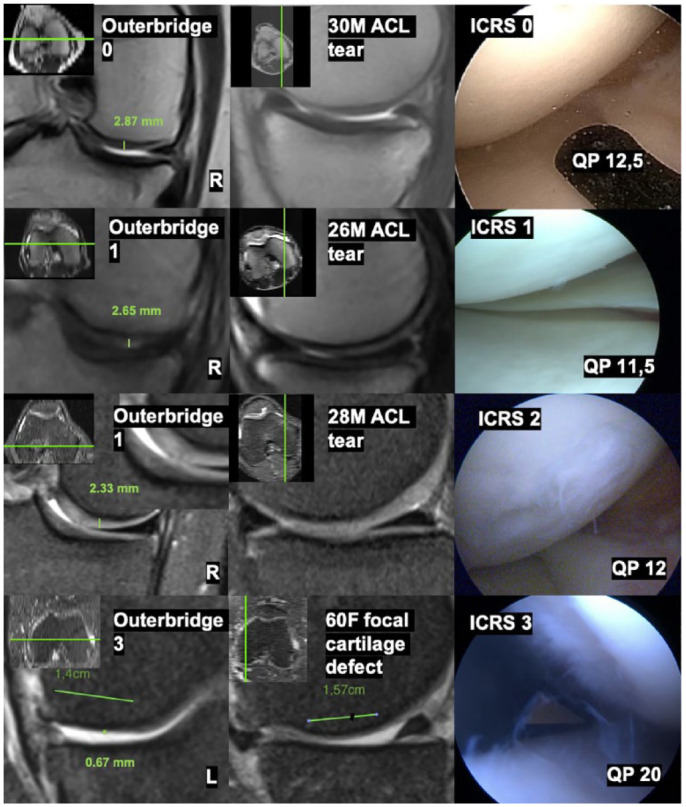
Reference picture of individual patients per ICRS grades 0 to 3 groups with demographics, pathology, 1.5 Tesla MRI images, cartilage thickness, Outerbridge grades, arthroscopic images, appointed ICRS grades, and QP values. ICRS = International Cartilage Repair Society; QP = quantitative parameter; MRI = magnetic resonance imaging.

Random patients per ICRS groups 0 to 3 are presented in reference to MRI images, Outerbridge grade, cartilage thickness, arthroscopic images, ICRS grade, QP values, patient demographics, and pathology (**Fig. 5**).

## Discussion

There are a number of studies that demonstrate the reliability of electromechanical assessment of articular cartilage *ex vivo* in comparison with histological and mechanical evaluation.^9,11,22,23^ Unfortunately, most of these studies were done on animal or *postmortem* human tissues. Our study performed on patients is directed toward clinical development of intraoperative arthroscopic cartilage evaluation.

The QP values of healthy, slightly altered, or severely damaged human articular cartilage of femoral condyles obtained during arthroscopic procedures were similar, compared with previously published *ex vivo* human studies.^9,12,13^ Sim *et al.*,^9^ in an *ex vivo* study of human femoral surfaces, reported healthy and slightly altered cartilage QP ranging from 7 to 11 that corresponds to Mankin scores of 0 to 2, fibril modulus ranging from 20+ to 10 MPa, and GAG content between 68 and ~28 ug/mg. More severe cartilage degeneration, corresponding to Mankin scores of 2 to 5, with more pronounced GAG and fibril modulus decrease was defined by QP ranging from 11 to 15.^9^ Mankin scores strongly correlate with ICRS grades; Spearman’s correlation coefficient of 0.977 was reported using cartilage-bone blocks obtained from knee arthroplasties.^24^ Another study performed on distal femoral condyles distinguished normal and abnormal cartilage, providing mean QP values of 8 for healthy ICRS grade 0, while it was QP of 14 for regions defined as cartilage degeneration (ICRS > 0).^12^ Another study by Sim *et al.*^13^ using 200 samples from 40 cadaveric femoral condyles described ICRS grade 0 and 1 corresponding to mean QP values of 7 and 12, respectively, while QP of 16 was attributed to ICRS grade 2.

The threshold between QP values of healthy and altered cartilage in the previously discussed studies ranges from 11 to 12. In our study, lower ICRS grades, which do not require cartilage reconstruction procedures, had QP values of 11.56 ± 2.3 SD, suggesting similar threshold values. The QP measurements obtained during knee arthroscopic surgery, corresponding to ICRS grades 0 to 2 of medial femoral condyle central weight-bearing surface state, were 10.5 (±2.8 SD), 12 (±0.7 SD), and 13.25 (±1.77 SD), respectively. No significant lower intergroup differences were detected, given uneven and modest group sizes. Nevertheless, detecting QP statistical differences among different ICRS grades is problematic and there are data suggesting difficult reproducibility of ICRS grading to distinguish between minor injury grades.^25^ If ICRS grading cannot reliably distinguish low-grade changes, then grouping QP values based on ICRS grades may confound electromechanical results among lower ICRS grades. Most recently Ukai *et al.*^14^ performed electromechanical assessment of human femoral head articular cartilage and observed no QP differences between ICRS grades 1 and 2. However, in our study, only a few MRI images of random patients with ICRS grades 0 to 2 injuries were gathered and presented in reference to macroscopic and QP values. Once again, it shows comparable electromechanical values between minor ICRS, Outerbridge grades, and QP values, although all patients were young men treated for ACL rupture.

Moreover, differences between healthy individuals exist. In our study, both patients with ICRS grade 0 cartilage had distant QP values of 7 ±0 SD, for the 36 F treated for menisci rupture, and 12.5 ±0.5 SD, for the 30 M treated for ACL tear. Of note, the slightly older woman had better QP results. Previously, no significant effect of smoking, body mass index, age, or gender on cartilage electromechanical properties were found.^13^ These findings suggest differences of cartilage electromechanical properties and bio-composition from individual to individual. This was reported in the literature as variance of interindividual GAG content.^26^ Possibly, inflammatory joint environment or physical activity could alter bio-histochemical cartilage structure as well.

The anatomical region and site may influence QP values. Representative QP mapping study revealed differences in QP measurements obtained from healthy human femoral cartilage regions and even greater differences between tibial and femoral surfaces.^12^ This disparity was also reported in the study using human cadaveric tibial plateaus, where a slightly changed ICRS grade 1 had QP of 18 (±3.3 SD), while cartilage injury, up to 50% of depth, corresponding to ICRS grade 2 had QP of 25.6 (±3 SD).^27^ Greater QP values related to the same ICRS scores were observed on tibial plateau, compared with our clinical and *ex vivo* femoral studies in the literature.^9,12,13^ Therefore, we have chosen a specific location for evaluation—medial femoral condyle weight-bearing central site. The QP value reference to a specific site is essential for clinical interpretation of electromechanical data.

Advanced, greater than ICRS grade 2, cartilage injury was characterized by QP greater than 15.^9^ In another, already discussed, paper, ICRS grade 3 cartilage injury had a mean QP value of 20.^13^ Our findings are numerically comparable to previously discussed *ex vivo* values, despite the fact they were gathered specifically in the medial femoral condyle weight-bearing central region. In the literature, no correlation was reported between electromechanical QP and cartilage thickness measured by a calibrated dissection microscope.^9^ The indenter force was previously determined to be independent from cartilage thickness within the range from 2 to 4 mm.^28^ In contrast, a reverse phenomenon of numerically lesser, therefore clinically superior, QP values is described due to cartilage thinning observed when injuries were greater than ICRS grade 3.^13^ This indicates that injuries through the subchondral bone, with no cartilage, corresponding to ICRS grade 4, cannot be assessed electromechanically. However, it was not observed during *in vivo* electromechanical study of femoral head surfaces, which reported worse electromechanical values in ICRS grade 4 group.^14^ We also report worse QP values of 30.17 (±2.3 SD) with ICRS grade 4. However, to avoid the described issue, measurements on the edge of the subchondral defect were taken.

A strong positive correlation between ICRS grades and QP values is reported. To our knowledge, there are no previous studies on electromechanical cartilage evaluation during knee arthroscopies with which we can directly compare our findings. In a previously published technical note, arthroscopic electromechanical assessment of healthy articular knee cartilage was reported.^19^ A QP value of 14.29 (±4.82 SD) was attributed to healthy femoral cartilage, which was numerically slightly greater, compared with our current findings and results from *ex vivo* studies. However, the QP value of the mentioned study is a mean of all healthy regions of the medial femoral condyle in comparison with specific central-central weight-bearing surface that we measured. The cartilage region prone to highest weight-bearing forces might have greater ECM content and therefore better QP values. Increased proteoglycan content in the cartilage was reported after applying cyclical loading to metacarpophalangeal joints of the rabbit.^29^ Furthermore, in our study, the ICRS grade 0 healthy cartilage group comprised young individuals (33.5 years ± 5.5 SD), while demographics of patients from reference study are not available for analysis. A comparison of electromechanical, macroscopic, and histological properties in a symptomatic human articular cartilage of the hip was reported by Ukai *et al.*^14^ However, we cannot discuss the results of this study as it had inversely proportional electromechanical values presented. Some literature reported electromechanical properties as streaming potential integral (SPI), which is inversely proportional to QP.^11,22^ The use of QP is supported in the literature due to its advantage over SPI regarding robustness to noise and simplicity.^9^

There are several drawbacks using this technique in the clinical setting. First, it takes numerous repetitions until QP value is obtained because the tip of the probe must be positioned precisely perpendicularly to the cartilage surface. The qualification and experience of the orthopedic surgeon allows to reduce the number of measurement attempts. Second, assistance from support staff is required because recording of the measurement using software and arthroscopic tip manipulation by the surgeon must be well coordinated to obtain a numeric value. Furthermore, additional sterile draping, preparation of isoelectric box, and software calibration before every measurement take significant amount of time.

Nevertheless, there is a need for further implementation of electromechanical evaluation method to advance objective intraoperative cartilage injury assessment techniques. Arthroscopically obtained QP values correlate with ICRS injury scores, suggesting that this technique could be readily applied and to differentiate between lower and greater ICRS grades when cartilage reconstruction is indicated. The electromechanical technique could be used clinically to determine the margins of cartilage debridement, evaluate cartilage under the conditions of osteochondritis dissecans or subchondral bone edema, when cartilage appears normal, evaluate the success of cartilage reconstruction procedures at second look, or aid the surgeon when the decision is not clear. Moreover, objective electromechanical method, representing cartilage biocomposition could be included as an additional cartilage evaluation method in the clinical studies. However, additional data are required to form accurate interpretation guidelines of intraoperative QP values, considering that cartilage electromechanical properties vary by site, region, and between individuals.

The major limitation of our study is the small and uneven sample size per ICRS injury group and evaluation of ICRS grade 4 cartilage injuries using a different approach. Another limitation could be attributed to the study design as only a few MRIs were obtained during the study and no statistical comparison between Outerbridge scores, or cartilage thickness, could have been made.

## Conclusion

Arthroscopically established electromechanical QP values of healthy and damaged femoral cartilage central weight-bearing surface correlate with the grade of visual ICRS injury and have similar numerical results compared with the findings of *ex vivo* studies. The QP values depicting minor cartilage changes of ICRS injury grades 0 to 2 were significantly lesser compared with greater ICRS injury grades of 3 and 4, requiring cartilage reconstruction. Our study suggests that cartilage deterioration could be fairly evaluated using intraoperative arthroscopic electromechanical evaluation.
